# State Estimation Using Dependent Evidence Fusion: Application to Acoustic Resonance-Based Liquid Level Measurement

**DOI:** 10.3390/s17040924

**Published:** 2017-04-21

**Authors:** Xiaobin Xu, Zhenghui Li, Guo Li, Zhe Zhou

**Affiliations:** 1 School of Automation, Hangzhou Dianzi University, Hangzhou 310018, China; 151060063@hdu.edu.cn (Z.L.); 162060112@hdu.edu.cn (G.L.); 2 School of Engineering, Huzhou University, Huzhou 310027, China; zzhou@zjhu.edu.cn

**Keywords:** DS evidence theory, state estimation, liquid level measurement, alarm monitoring

## Abstract

Estimating the state of a dynamic system via noisy sensor measurement is a common problem in sensor methods and applications. Most state estimation methods assume that measurement noise and state perturbations can be modeled as random variables with known statistical properties. However in some practical applications, engineers can only get the range of noises, instead of the precise statistical distributions. Hence, in the framework of Dempster-Shafer (DS) evidence theory, a novel state estimatation method by fusing dependent evidence generated from state equation, observation equation and the actual observations of the system states considering bounded noises is presented. It can be iteratively implemented to provide state estimation values calculated from fusion results at every time step. Finally, the proposed method is applied to a low-frequency acoustic resonance level gauge to obtain high-accuracy measurement results.

## 1. Introduction

Estimating the state of a dynamic system based on noisy sensor measurements is a common problem in sensor methods and applications [1,2]. Mainstream estimation methods all assume that both the system state noise and measurement noise can be modeled as random variables with known statistical properties. The Kalman filter, which supposes both of noises obey Gaussian distributions, is, by far, the most popular method [3]. The basic Kalman filter is only applicable to linear systems. In order to deal with nonlinear cases Bucy and Sunahara proposed the extended Kalman filter (EKF) [4,5]. The EKF uses the first order Taylor expansion technique to linearize state and observation equations, and then obtains state estimations by the Kalman filter. On the other hand, approximation to a state probability distribution of a nonlinear system is, to a great extent, easier and more feasible than a linear approximation to a nonlinear function [6]. Based on this idea, Gordon and Salmond proposed the particle filter (PF) [6]. The performance of the PF is commonly superior to the EKF because it can usually provide more precise information about state posterior probability distribution than does the EKF, especially when it takes a multimodal shape or noise distributions are non-Gaussian [6,7].

The precondition of the above methods is that the noise statistical properties must be known. However, in some practical applications, what engineers can obtain are not precise statistical distributions [8], but ranges of noises. Hence, a group of state estimation methods considering bounded noises, also known as the bounded-error methods, appeared [9,10,11,12]. Assuming that all variables belong to known compact sets, these methods build simple sets, such as ellipsoids or boxes, guaranteed to contain all state vectors consistent with given constraints. For linear systems, some scholars began to study such state estimation methods in the 1960s [9,10,11]. For nonlinear systems, the corresponding studies are relatively rare. Khemane et al. and Jaulin proposed bounded-error state and parameter estimations for nonlinear systems [13,14]. Gning proposed a relatively simple and fast bounded-error method based on interval analysis and constraint propagation, which was successfully applied to dynamic vehicle localization [12], but when the noise bounds cannot be precisely determined, its robustness will unavoidably decline [7]. That is to say, if the bounds are too tight, then the data may become inconsistent with the system equations, and in this case, this method fails to provide a solution. On the contrary, if the bounds are overestimated, then the estimated state becomes very imprecise, and this method becomes overly pessimistic [7].

In order to deal with this problem, Nassreddine proposed an improved method by integrating interval analysis with DS evidence theory. Its key idea is to replace the set-based representation of uncertainty by a more general formalism, namely, mass functions in evidence theory [7]. It introduces possibility distributions to model bounded noises, and then uses mass functions, i.e., evidence composed of interval focal elements and their masses to approximate these distributions. Essentially, such mass functions can be regarded as “generalized boxes” composed of a collection of boxes with associated weights. These mass functions can be propagated in the system equations using interval arithmetic and constraint-satisfaction techniques to get the mass function of system state at each time step. Pignistic expectation of this mass function is calculated as the state estimation value. Therefore, this approach extends the pure interval approach, making it more robust and accurate.

Nassreddine’s research showed the powerful ability of DS evidence theory to deal with the uncertainty of dynamic systems. Hence, this paper further presents a new state estimation method, which uses not only evidential description of uncertainty, but also dependent evidence fusion. Here, the state equation and the observation equation of a dynamic system and the actual observations of system states are regarded as three information sources. The random set description of evidence and extension principle of random set is used to obtain state evidence and observation evidence from these three information sources and to propagate them in the system equations. There are correlation among these evidence, so the proposed combination rule of dependent evidence is used to fuse the propagated evidence and Pignistic expectation of fusion results is calculated as state estimation value at each time step. Compared with Nassreddine’s method, it is shown that the proposed approach generates more accurate estimation results by combining dependent evidence. An industrial liquid level detection apparatus was employed to show the better performance of the approach.

## 2. Foundations of Dempster-Shafer (DS) Evidence Theory

The DS theory is a mechanism formalized by Shafer for representing and reasoning with uncertain, imprecise, and incomplete information. It is initially based on Dempster’s original work on the modeling of uncertainty in terms of upper and lower probabilities induced by a multi-valued mapping rather than as a single probability value [15]. One of the specificities of this theory is that the objects of study are no more the universe, i.e., a set, defined as the frame of discernment hereinafter, but the power set of this universe. In this section we introduce some main concepts of this theory and some necessary notions that will be used in the proposed approach. A more detailed exposition and some background information can be found in [16].

### 2.1. Basic Concepts in DS Evidence Theory

**Definition** **1** (Frame of discernment)**.** A set is called a frame of discernment if it contains mutually exclusive and exhaustive possible hypotheses. This set is usually denoted as Θ. The power set of Θ is denoted as 2^Θ^.

**Definition** **2** (Mass function)**.** A function m: 2^Θ^ → [0, 1] is called a mass function on Θ if it satisfies the following two conditions: (1) m(∅)=0; (2) $\sum_{A\in 2^{\Theta }}m(A)=1$. This function is also named as a basic belief assignment (BBA). A subset A with a non-null mass is viewed as a focal element. Commonly, if an information source can provide a mass function on Θ, then this mass function is called a body of evidence, abbreviated to evidence (E).

**Definition** **3** (Dempster’s combination rule)**.** *If m_*1*_, m_*2*_ are two BBAs induced from two statistically independent information sources, then a combined BBA can be obtained by using Dempster’s combination rule:*

(1) $$m(A)=\{\begin{array}{cc} \frac{\sum_{B\cap C=A}m_{1}(B)m_{2}(C)}{1-\sum_{B\cap C=\emptyset }m_{1}(B)m_{2}(C)}, & A\subseteq \Theta \;and\;A\neq \emptyset \\ 0, & A=\emptyset \end{array}$$

*Note that the Dempster’s combination rule is meaningful only when* $\sum_{B\cap C=\emptyset }m_{1}(B)m_{2}(C)<1$ *, i.e., m*_1_ *and m*_2_ *are not totally conflicting. This rule can be used to synthesize uncertain, imprecise or incomplete information coming from different sources.*

### 2.2. The Degree of Dependence and the Combination of Dependent Evidence

In DS evidence theory, Dempster’s combination rule is the most important tool for computing a new BBA from two BBAs based on two pieces of evidence. This rule requires that the two pieces of evidence must be independent, which is considered to be a very strong constraint and cannot always be met in practice. Wu, Yang and Liu [17] pointed that if there are two pieces of evidence which are partially derived from the same information source, then both of them are mutually dependent. This interpretation concentrates on the connotation of independence conception in evidence combination operation. In this case, Wu, Yang and Liu [17] proposed the energy of evidence concept, and then, deduced the degree of dependence and the dependency coefficient between the two from the energy of the intersection of the two. Based on these notions, the combination of dependent evidence can be realized.

**Definition** **4** (The energy of evidence E)**.** *The energy of evidence E, En(E) is defined as:*

(2) $$\mathrm{En}(E)=\sum_{\begin{array}{l} i=1\\ A_{i}\neq \Theta \end{array}}^{n(E)}\frac{m(A_{i})}{\left|A_{i}\right|}$$

*where |A_i_| is the number of elements in the focal element A_i_, n(E) is the number of distinct focal elements in E. Obviously, En(E) have some valuable characteristics: (1) if every m(A_i_) = 0, namely, m(Θ) = 1, then En(E) = 0 and the evidence E represents no useful information; (2) if |A_i_| = 1 and m(Θ) = 0, then En(E) = 1 and the E contains the maximum useful information; (3) En(E) ∈ [0, 1].*

*Suppose that the BBAs of evidence E*_1_ *and E*_2_ *are m*_1_ *and* *m*_2_*, respectively, and their focal element sequences are A_i_ and B_j_. It is possible that some focal elements of E*_1_ *and E*_2_ *are induced by the same information source. In this case, E*_1_ *and* *E*_2_ *will be dependent, then the energy of the intersection of the two pieces of evidence can be described by:*

(3) $$\mathrm{En}(E_{1},E_{2})=\sum_{\begin{array}{l} ij=1\\ D_{ij}\neq \emptyset \end{array}}^{\left|\{D_{ij}\}\right|}\frac{m(D_{ij})}{\left|D_{ij}\right|}$$

*where* *D_ij_* *denotes dependent focal element, |{D_ij_}| is the number of distinct D_ij_ and the BBA function m is derived from m*_1_ *and* *m*_2_*.*

**Figure 1:**
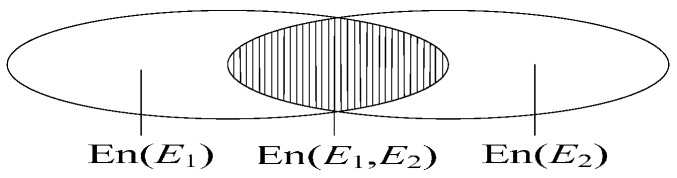
The relationships of two pieces of dependent evidence.

The relationship of En(*E*_1_), En(*E*_2_) and En(*E*_1_, *E*_2_) is illustrated in Figure 1; especially, En(*E*_1_, *E*_2_) = 0 implies the independence between *E*_1_ and *E*_2_. The value of En(*E*_1_, *E*_2_) measures the dependency of the two pieces of evidence.

**Definition** **5** (The degree of dependence between two pieces of evidence)**.** *En(E_*1*_, E_*2*_) is defined as the degree of dependence between E_*1*_ and E_*2*_. Actually, the partial energies En(E_*1*_) − En(E_*1*_, E_*2*_) in E_*1*_ and En(E*_2_*) − En(E_*1*_, E_*2*_) in E_*2*_ are independent of each other. If energy En(E_*1*_, E_*2*_) is partitioned into two parts, with each part attached to E_*1*_ and E_*2*_, respectively, as follows:*

(4) $$D(E_{1},E_{2})=\frac{2\mathrm{En}(E_{1},E_{2})}{\mathrm{En}(E_{1})+\mathrm{En}(E_{2})}$$

*then two corresponding independent pieces of evidence can be generated from* *E*_1_ *and* *E*_2_*.*

*For* *E*_1_*, its final independent energy can be calculated as:*

(5) $$\begin{array}{ll} {\mathrm{En}}_{f}(E_{1}) & =\mathrm{En}(E_{1})-\mathrm{En}(E_{1},E_{2})+\mathrm{En}(E_{1},E_{2})\frac{\mathrm{En}(E_{1})}{\mathrm{En}(E_{1})+\mathrm{En}(E_{2})}\\ & =\mathrm{En}(E_{1})-\mathrm{En}(E_{1},E_{2})\frac{\mathrm{En}(E_{2})}{\mathrm{En}(E_{1})+\mathrm{En}(E_{2})}\\ & =\mathrm{En}(E_{1})\left(1-\frac{\mathrm{En}(E_{1},E_{2})}{\mathrm{En}(E_{1})+\mathrm{En}(E_{2})}*\frac{\mathrm{En}(E_{2})}{\mathrm{En}(E_{1})}\right)\\ & =\mathrm{En}(E_{1})\left(1-\frac{1}{2}D(E_{1},E_{2})*\frac{\mathrm{En}(E_{2})}{\mathrm{En}(E_{1})}\right) \end{array}$$

*Similarly:*

(6) $${\mathrm{En}}_{f}(E_{2})=\mathrm{En}(E_{2})\left(1-\frac{1}{2}D(E_{1},E_{2})*\frac{\mathrm{En}(E_{1})}{\mathrm{En}(E_{2})}\right)$$

**Definition** **6** (The dependency coefficient between two pieces of evidence)**.** *The dependency coefficient of E*_1_ *to E*_2_ *is defined as:*

(7) $$R_{12}=\frac{1}{2}D(E_{1},E_{2})\frac{\mathrm{En}(E_{2})}{\mathrm{En}(E_{1})}$$

*and the dependency coefficient of* *E*_2_ *to* *E*_1_ *is defined as:*

(8) $$R_{21}=\frac{1}{2}D(E_{1},E_{2})\frac{\mathrm{En}(E_{1})}{\mathrm{En}(E_{2})}$$

*E*_1_ *and* *E*_2_ *can be modified by* *R*_12_ *and* *R*_21_*, respectively, to obtain their corresponding independent* *E*_1_*′* *and* *E*_2_*′, their BBA functions are given by:*

(9) $${m_{1}}^{\prime }(A)=\left\{ \begin{array}{ll} (1-R_{12})m_{1}(A), & \forall A\subseteq \Theta ,A\neq \Theta \\ 1-\sum_{A\subset \Theta }{m_{1}}^{\prime }(A) & A=\Theta \end{array}\right.$$

(10) $${m_{2}}^{\prime }(B)=\left\{ \begin{array}{ll} (1-R_{21})m_{2}(B), & \forall B\subseteq \Theta ,B\neq \Theta \\ 1-\sum_{B\subset \Theta }{m_{2}}^{\prime }(B) & B=\Theta \end{array}\right.$$

*Consequently, the requirement of Dempster’s rule is met and the combination of E*_1_*′ and E*_2_*′ can be implemented according to Dempster’s rule in (1). Finally, the combination of E*_1_ *and E*_2_ *is indirectly realized by the combination of E*_1_*′ and E*_2_*′. Actually, reference* [17] *gives the decorrelation method to correct E*_1_ *and E*_2_ *by dependency coefficients such that the corrected E*_1_*′ and E*_2_*′ can be deemed as the independent evidence and combined using Dempster’s rule.*

### 2.3. The Random Set Description of Evidence

#### 2.3.1. Random Set and Random Relation

**Definition** **7** (Random set [18,19])**.** *A finite support random set on Θ is a pair (ℱ,m) where ℱ is a finite family of distinct non-empty subsets of Θ and m is a mapping ℱ → [0, 1] and such that ∑_A__∈__ℱ_m(A) = 1.*

ℱ is called the support of the random set and m is called a basic belief assignment. Such a random set (ℱ,m) is equivalent to a mass function in the sense of Shafer.

**Definition** **8** (Random relation [18,19])**.** *Let Θ = Θ*_1_ *× Θ*_2_ *× …× Θ_n_ be a multi-dimensional space, where “×” indicates Cartesian product. A finite support random relation is a random set (ℱ,m) on Θ.*

*The projections of a random relation on Cartesian product Θ*_1_ *× Θ*_2_ *×…× Θ_n_ are defined by Shafer to be the marginal random set (ℱ_k_,m_k_) (k = 1,2,…, n):*

(11) $$\forall C_{k}\subseteq \Theta_{k},m_{k}(C_{k})=\sum \left\{m(A)|C_{k}={\mathrm{Proj}}_{\Theta_{k}}(A)\right\}$$

(12) $${\mathrm{Proj}}_{\Theta_{k}}(A)=\{u_{k}\in \Theta_{k}|\exists u=(u_{1},\cdots ,u_{k},\cdots u_{n})\in A\}$$

*For* ∀A∈ℱ *, A = C*_1_ *× C*_2_ *×…× C_n_, if m(A) = m*_1_*(C*_1_*) × m*_2_*(C*_2_*) ×…× m_n_(C_n_), then (ℱ,m) is called decomposable Cartesian product random relation, and marginal random sets (ℱ*_1_*,m*_1_ *), (ℱ*_2_*,m*_2_*),…, (ℱ_n_,m_n_) are mutually independent.*

#### 2.3.2. Extension Principles

Let *ξ* = (*ξ*_1_, *ξ*_2_,…, *ξ_n_*) be a variable on Θ = Θ_1_ × Θ_2_ × …× Θ*_n_*, *ζ = f*(*ξ*), ζ∈Φ, *f* : Θ→Φ is the function of *ξ*. The random set (ℛ,*ρ*) of *ζ*, which is the image of random relation (ℱ,*m*) of *ξ* through *f*, is given by extension principles [20,21,22]:

(13) $$R=\left\{R_{i}=f(A_{i})|A_{i}\in F\right\}$$

(14) $$\rho (R_{j})=\sum \left\{m(A_{i})|R_{j}=f(A_{i})\right\}$$

where:

(15) $$f(A_{i})=\{f(u)|u\in A_{i}\},i=1,2,\cdots ,M$$

*M* is the number of element of ℱ. The summation in Equation (14) accounts for the fact that more than one focal element *A_i_* may yield the same image *R_j_* through *f*.

The key of constructing (ℛ,*ρ*) is to calculate the image of *A_i_* through *f*. If *ξ* is a continuous variable on Θ, then Θ = ℝ*^n^*, ℱ becomes a finite family of distinct non-empty sub-intervals on Θ. In this case, the process of constructing (ℛ,*ρ*) is given as follows:

For each *ξ_k_* in *ξ*, let its marginal random set be (ℱ*_k_*,*m_k_*) and the focal element of (ℱ*_k_*,*m_k_*) be a interval [*a_k_^-^*,*a_k_^+^*], then the focal element of (ℱ,*m*) can be given as:

(16) $$A=[a_{1}^{-},a_{1}^{+}]\times \cdots \times [a_{n}^{-},a_{n}^{+}]$$

The image of *A* can be calculated by using the methods of Interval Analysis [19,20,21]; if *A* is a convex set, then *A* has 2*^n^* vertices, denoted as *v_j_* (*j* = 1,2,…, 2*^n^*). If function *f* has certain properties, the Vertex Method can help reduce the calculation time considerably [22]:

**Proposition** **1.** ∀A∈ℱ *, if ζ = f(ξ) is continuous in A and also no extreme point exists in this region (including its boundaries), then the value of interval function can be obtained by:*

(17) $$f(A)=R=[\underset{j}{\min}\{f(v_{j}):j=1,2,\cdots ,2^{n}\},\underset{j}{\max}\{f(v_{j}):j=1,2,\cdots ,2^{n}\}]$$

*Thus, function f has to be evaluated 2^n^ times for each focal element A. This computational burden can be further reduced if the hypotheses of the following Proposition 2 hold [21].*

**Proposition** **2.** *If f is continuous, if its partial derivatives are also continuous and if f is a strictly monotonic function with respect to each ξ_k_, k = 1, 2,…, n, then:*

(18) $$\exists \;v_{\min},\;f(v_{\min})=\underset{j}{\min}\{f(v_{j}):j=1,2,\cdots ,2^{n}\}$$

(19) $$\exists \;v_{\max},\;f(v_{\max})=\underset{j}{\max}\{f(v_{j}):j=1,2,\cdots ,2^{n}\}$$

*There is a case in point. Let ξ = (ξ*_1_*,ξ*_2_*,ξ*_3_*), A = [a*_1_*^-^,a*_1_*^+^]* *× [a*_2_*^-^,a*_2_*^+^]* *× [a*_3_*^-^,a*_3_*^+^]. Assume f and its partial derivatives are all continuous. If f is increasing with respect to ξ*_1_* and ξ*_2_*, decreasing with respect to ξ*_3_ *respectively, then f has to be calculated only twice for each focal element A, namely, f(A) = [f(v_min_),f(v_max_)], v_min_ = [a*_*1*_*^-^,a*_*2*_*^-^,a*_*3*_*^+^], v_max_ = [a*_1_*^+^,a*_2_*^+^,a*_3_*^-^].* *Totally,* *2M evaluations* *of f are needed to obtain complete (ℛ,ρ).*

*Furthermore, the expectation of (ℛ,ρ) is given by [23]:*

(20) $$𝔼(\rho )=\sum_{j=1}^{N}\rho (R_{j})\cdot \left(\frac{r_{j}^{+}+r_{j}^{-}}{2}\right)$$

*where R_j_ = [r_j_^-^, r_j_^+^], j = 1,2,⋯, N, N is the number of focal element R_j_.*

## 3. State Estimation Based on Dependent Evidence Fusion

### 3.1. Dynamic System Model under Bounded Noises

**Figure 2:**
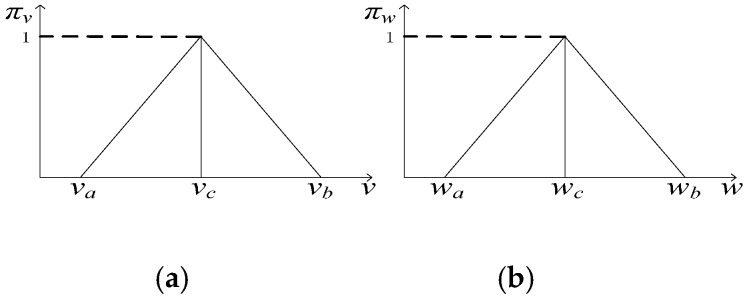
(**a**) Triangle possibility distributions of state noises, (**b**) Triangle possibility distributions of observation noises.

The dynamic systems mode constructed by the state and observation equations is as follows:

(21) $$\left\{ \begin{array}{l} x_{k+1}=f(x_{k},v_{k})\\ z_{k+1}=g(x_{k+1},w_{k+1}) \end{array}\right.k=1,2,3,\cdots$$

where the relationship between state *x_k+_*_1_ at time *k* + 1 and state *x_k_* at time *k* is described as function *f*. The relationship between observation *z_k+_*_1_ at time *k +* 1 and state *x_k+_*_1_ at time *k +* 1 is described as function *g*. *v_k_* and *w_k_* are bounded additive state noise variable and observation noise variable respectively, which are independent of each other. These two noises can be approximated to triangle possibility distributions [7], noted as π*_v_* and π*_w_*, respectively, (the noise distributions are identical at each time step), as shown in Figure 2.

(22) $$\pi_{v}(v)=\left\{ \begin{array}{ll} \frac{v-v_{a}}{v_{c}-v_{a}} & if\;v_{a}\leq v\leq v_{c}\\ \frac{v_{b}-v}{v_{b}-v_{c}} & if\;v_{c}\leq v\leq v_{b}\\ 0 & otherwise \end{array}\right.$$

where [*v_a_,v_b_*] is the support interval of the state noise, *v_c_* is the mode of state noise, similarly:

(23) $$\pi_{w}(w)=\left\{ \begin{array}{ll} \frac{w-w_{a}}{w_{c}-w_{a}} & if\;w_{a}\leq w\leq w_{c}\\ \frac{w_{b}-w}{w_{b}-w_{c}} & if\;w_{c}\leq w\leq w_{b}\\ 0 & otherwise \end{array}\right.$$

### 3.2. Recursive Algorithm of State Estimation Based on Extension Principles and Dependent Evidence Fusion

**Figure 3:**
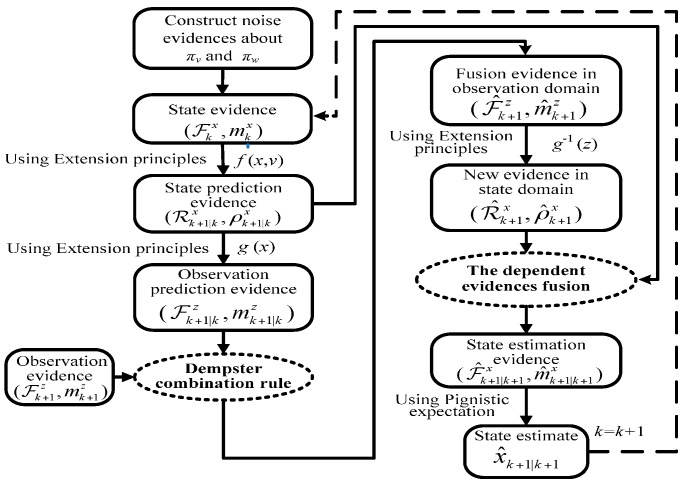
Flowchart of state estimation iterative algorithm.

Figure 3 shows the flow of the proposed recursive algorithm. The following steps will be introduced in detail.

**Step 1: Construct noise evidence to approximate π*_v_* and π*_w_***. Initially, we construct evidence $\left({ℱ}_{k}^{v},m_{k}^{v}\right)$ to approximate the possibility distribution *π_v_* of state noise variable *v_k_*. For any α ∈ (0, 1], α cut set of π*_v_* is [9]:

(24) $$\left[\pi_{\alpha }^{v-},\pi_{\alpha }^{v+}]=\{v|\pi_{v}(v)\geq \alpha \right\}$$

If there exist α_0_, α_1_,⋯, α*_p_*_−1_ which satisfy 0 = α_0_ < α_1_ <⋯< α*_p_*_−1_ < 1, then their corresponding α-cut sets will satisfy $\left[\pi_{\alpha_{0}}^{v-},\pi_{\alpha_{0}}^{v+}]\subset [\pi_{a_{1}}^{v-},\pi_{a_{1}}^{v+}]\subset \cdots \subset [\pi_{\alpha_{p-1}}^{v-},\pi_{\alpha_{p-1}}^{v+}\right]$ where *p* is a positive integer. Take these *p* α-cut sets as focal elements with nested closed interval forms, then their corresponding BBAs are:

(25) $$m([\pi_{\alpha_{i}}^{v-},\pi_{\alpha_{i}}^{v+}])=\{\begin{array}{c} \alpha_{i+1}\\ \alpha_{i+1}-\alpha_{i}\\ 1-\alpha_{i} \end{array}\;\begin{array}{c} if\;i=0\\ if\;i=1,2,\cdots ,p-2\\ if\;i=p-1 \end{array}$$

**Figure 4:**
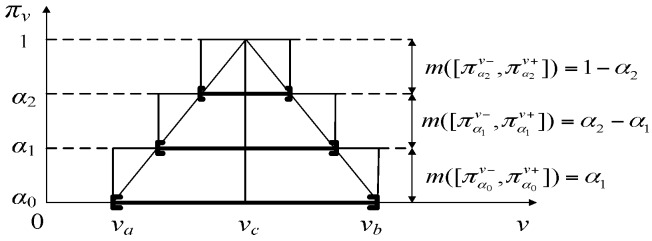
The possibility distribution of state noise and its evidence construction.

Figure 4 gives an example that when *p* = 3, and *m* can be constructed by uniformly cutting *α* three times. Distinctly, *m* corresponds to a possibility distribution π that approximates π*_v_*. Certainly, a better approximation of the continuous possibility distribution can be obtained by increasing the number *p* of cut sets, at the expense of higher computational complexity.

It is worth noticing that *m* is constructed on the condition that all values outside the support interval [*v_a_,v_b_*] are completely impossible. However, in practice, the bounds *v*_a_ and *v*_b_ are commonly given based on available measurement knowledge or real data, so they may not be precise and the values outside [*v_a_,v_b_*] may appear. To account for the imprecision of the support interval [7], constructs $\left({ℱ}_{k}^{v},m_{k}^{v}\right)$ by discounting *m* with a small discount rate *ε_v_*, in which, $m_{k}^{v}$ is defined as [9]:

(26) $$m_{k}^{v}(A)=\{\begin{array}{c} \left(1-\varepsilon_{v})m(A\right)\\ (1-\varepsilon_{v})m(\Theta )+\varepsilon_{v} \end{array}\;\begin{array}{c} if\;A\in \Xi \\ if\;A=\Theta \end{array}$$

where $\Xi =\{[\pi_{\alpha_{i}}^{v-},\pi_{\alpha_{i}}^{v+}]|i=1,2,\cdots ,p-1\}$, Θ = ℝ, accordingly, ${ℱ}_{k}^{v}=\Xi \cup \Theta$. In the course of implementing the proposed algorithm, Θ can be replaced by the closed interval [*v_a_*′,*v_b_*′], here *v_a_*′ >> *v_a_* and *v_b_*′ >> *v_b_* such that the following interval operations can be done easily.

In the same way, we can construct evidence $\left({ℱ}_{k}^{w},m_{k}^{w}\right)$ using the possibility distribution π_w_ of observation noise variable *w_k_*.

**Step 2: Obtain state prediction evidence**. $E_{k+1|k}^{x}=(R_{k+1|k}^{x},\rho_{k+1|k}^{x})$ **at time *k* + 1 from state equation**. Suppose the estimation result at time *k* is ${\hat{x}}_{k|k}$. When *k* = 1, ${\hat{x}}_{1|1}$ is initialized as real observation *z*_1_. Considering the influence of noise to the state, we construct the state evidence $\left({ℱ}_{k}^{x},m_{k}^{x}\right)$ of ${\hat{x}}_{k|k}$ by adding noise to ${\hat{x}}_{k|k}$:

$${ℱ}_{k}^{x}=\{[\pi_{\alpha_{0}}^{v-}+{\hat{x}}_{k|k},\pi_{\alpha_{0}}^{v+}+{\hat{x}}_{k|k}],[\pi_{a_{1}}^{v-}+{\hat{x}}_{k|k},\pi_{a_{1}}^{v+}+{\hat{x}}_{k|k}],\;\cdots ,[\pi_{\alpha_{q-1}}^{v-}+{\hat{x}}_{k|k},\pi_{\alpha_{q-1}}^{v+}+{\hat{x}}_{k|k}],\Theta \}\;;\;m_{k}^{x}=m_{k}^{v}$$

Thus, taking $\left({ℱ}_{k}^{v},m_{k}^{v}\right)$ and $\left({ℱ}_{k}^{x},m_{k}^{x}\right)$ as the inputs of state equation $x_{k+1}=f(x_{k},v_{k})$, we can get the state prediction evidence $E_{k+1|k}^{x}=({ℛ}_{k+1|k}^{x},\rho_{k+1|k}^{x})$ by mapping from the inputs to the outputs based on the extension principles in Equations (13) and (14).

**Step 3: Obtain observation prediction evidence**. $E_{k+1|k}^{z}=(F_{k+1|k}^{z},m_{k+1|k}^{z})$ at time *k* + 1 from observation equation.

Taking the state prediction evidence $\left({ℛ}_{k+1|k}^{x},\rho_{k+1|k}^{x}\right)$ in Step 2 as the input of the observation equation $g(x_{k+1})$, we can get $E_{k+1|k}^{z}=(F_{k+1|k}^{z},m_{k+1|k}^{z})$ based on the extension principles in (13) and (14).

**Step 4: Obtain fusion evidence**. ${\hat{E}}_{k+1}^{z}=({\hat{F}}_{k+1}^{z},{\hat{m}}_{k+1|k}^{z})$ at time *k* + 1 in observation domain.

Firstly, in Step 1, we get evidence $\left({ℱ}_{k}^{w},m_{k}^{w}\right)$ using the possibility distribution π_w_ of *w_k_*:

$${ℱ}_{k}^{w}=\{[\pi_{\alpha_{0}}^{w-},\pi_{\alpha_{0}}^{w+}],[\pi_{a_{1}}^{w-},\pi_{a_{1}}^{w+}],\cdots ,[\pi_{\alpha_{p-1}}^{w-},\pi_{\alpha_{p-1}}^{w+}],\Theta \}\;m_{k}^{w}=m_{k}^{v}$$

After getting observation *z*_k+1_ at time *k* + 1, considering the influence of noise to the observation, we construct the evidence $\left({ℱ}_{k+1}^{z},m_{k+1}^{z}\right)$ of *z_k_*_+1_ through adding noise to *z_k_*_+1_:

$${ℱ}_{k+1}^{z}=\{[\pi_{\alpha_{0}}^{w-}+z_{k+1},\pi_{\alpha_{0}}^{w+}+z_{k+1}],[\pi_{a_{1}}^{w-}+z_{k+1},\pi_{a_{1}}^{w+}+z_{k+1}],\cdots ,[\pi_{\alpha_{p-1}}^{w-}+z_{k+1},\pi_{\alpha_{p-1}}^{w+}+z_{k+1}],\Theta \}\;;\;m_{k}^{z}=m_{k}^{w}.$$

Secondly, using Dempster′s combination rule, we fuse $\left({ℱ}_{k+1}^{z},m_{k+1}^{z}\right)$ and $\left({ℱ}_{k+1|k}^{z},m_{k+1|k}^{z}\right)$ to get the fusion evidence ${\hat{E}}_{k+1}^{z}=({\hat{F}}_{k+1}^{z},{\hat{m}}_{k+1}^{z})$ in observation domain at time *k* + 1. As for the relationship between $\left({ℱ}_{k+1|k}^{z},m_{k+1|k}^{z}\right)$ and $\left({ℱ}_{k+1}^{z},m_{k+1}^{z}\right)$. The former is obtained by propagating ${\hat{x}}_{k|k}$ from state equation $f(x_{k},v_{k})$ to observation equation $g(x_{k+1})$; the latter is constructed by adding noise $\pi_{w}(w)$ to *z_k_*_+1_. It can be seen that the former completely comes from the state information ${\hat{x}}_{k|k}$ at past time step *k* which does not use the observation noise $w_{k+1}$($\pi_{w}(w)$), but uses the state noise $v_{k}$($\pi_{v}(v)$). Because $w_{k+1}$($\pi_{w}(w)$) and $v_{k}$($\pi_{v}(v)$) are independent of each other, so it is believed that the former and the latter are also independent of each other. Hence both of them can be directly fused using Dempster′s combination rule.

**Step 5: Get new evidence**. ${\hat{E}}_{k+1}^{x}=({\hat{ℛ}}_{k+1}^{x},{\hat{\rho }}_{k+1}^{x})$ at time *k* + 1 in state domain.

Taking ${\hat{ℱ}}_{k+1}^{z},{\hat{m}}_{k+1}^{z}$ attained in the Step 4 as the input of inverse function $g^{-1}(z_{k+1})$, we can get $\left({\hat{ℛ}}_{k+1}^{x},{\hat{\rho }}_{k+1}^{x}\right)$ by using the extension principles in Equations (13) and (14).

**Step 6: Get state estimation evidence**. $\left({\hat{ℱ}}_{k+1|k+1}^{x},{\hat{m}}_{k+1|k+1}^{x}\right)$ and state estimate ${\hat{x}}_{k+1|k+1}$ at time *k* + 1.

Using Dempster′s combination rule, we can fuse $\left({ℛ}_{k+1}^{x},\rho_{k+1}^{x}\right)$ attained in Step 5 and $\left({ℛ}_{k+1|k}^{x},\rho_{k+1|k}^{x}\right)$ attained in Step 2. That is to say, we utilize the former to revise the latter to get state estimation evidence $\left({\hat{ℱ}}_{k+1|k+1}^{x},{\hat{m}}_{k+1|k+1}^{x}\right)$. $\left({\hat{ℛ}}_{k+1}^{x},{\hat{\rho }}_{k+1}^{x}\right)$ is obtained by inverse mapping of fusion evidence $\left({\hat{ℱ}}_{k+1}^{z},{\hat{m}}_{k+1}^{z}\right)$ in observation domain. $\left({\hat{ℱ}}_{k+1}^{z},{\hat{m}}_{k+1}^{z}\right)$ is obtained by the fusion of observation evidence $\left({ℱ}_{k+1}^{z},m_{k+1}^{z}\right)$ and observation prediction evidence $\left({ℱ}_{k+1|k}^{z},m_{k+1|k}^{z}\right)$. In Step 3, it is noted that $\left({ℱ}_{k+1|k}^{z},m_{k+1|k}^{z}\right)$ is related to $\left({ℛ}_{k+1|k}^{x},\rho_{k+1|k}^{x}\right)$, so $\left({\hat{ℛ}}_{k+1}^{x},{\hat{\rho }}_{k+1}^{x}\right)$ and $\left({ℛ}_{k+1|k}^{x},\rho_{k+1|k}^{x}\right)$ are certainly mutually dependent. Therefore, the combination of dependent evidence must be used for fusing both of them. For the focal elements of $\left({\hat{ℛ}}_{k+1}^{x},{\hat{\rho }}_{k+1}^{x}\right)$ and $\left({ℛ}_{k+1|k}^{x},\rho_{k+1|k}^{x}\right)$ are the closed intervals on real numbers, here we extend the combination of dependent evidence in the discrete frame of discernment introduced in Section 2.2 to that in the continuous frame of discernment (see the corresponding proposition and example in Appendix A). $\left({\hat{ℛ}}_{k+1}^{x},{\hat{\rho }}_{k+1}^{x}\right)$ and $\left({ℛ}_{k+1|k}^{x},\rho_{k+1|k}^{x}\right)$ can be fused using the extended combination of dependent evidence to get state estimation evidence $\left({\hat{ℱ}}_{k+1|k+1}^{x},{\hat{m}}_{k+1|k+1}^{x}\right)$ at time *k +* 1.

Finally, Pignistic expectation of $\left({\hat{ℱ}}_{k+1|k+1}^{x},{\hat{m}}_{k+1|k+1}^{x}\right)$ is calculated as state estimation value ${\hat{x}}_{k+1|k+1}$ by Equation (20). Using state estimation at time *k* + 1 to do next iteration, we can estimate state at every time step.

In conclusion, as shown in Figure 3, the whole recursive algorithm is actualized under the framework of DS evidence theory. The corresponding evidence in state and observation domains are not only propagated and transformed by the extension principle, but also fused by the Dempster′s combination rule and the proposed combination rule for dependent evidence. Especially, fusion procedure can make that the masses focus to those interval focal elements that contain the system state, so as to get the accurate estimation results, which is the main difference from Nassreddine’s method under the framework of the interval analysis. In next section, our approach will be applied to liquid level estimation using an industrial level apparatus to show its better performance than possible with Nassreddine’s method.

## 4. Application to Liquid Level Measurement

Level measurement methods based on sound reflection phenomena have been successfully applied in some areas of process industry (chemical, waste water treatment, petroleum, etc.) because the level is the main monitored process variable used in industrial alarm systems. Ultrasonic measurement methods, with good directivity, convenient operation and so on, have become some of the most commonly used techniques [24]. Their measuring principle is to emit an ultrasound toward a liquid surface and receive the echos, then to calculate the distance from the surface to the acoustic receiver device by multiplying the sound velocity by the round-trip time [25]. However, this method is susceptible to the quality of the instrument itself and environmental noise, which will deteriorate the measurement accuracy. Besides, if the ultrasound encounters foams, residues, deposits, etc., in the measurement process, it is also prone to parasitic reflection, thereby the ultrasound propagation path is changed, which seriously affects the measurement accuracy [26].

On the contrary, low-frequency sound waves have longer wavelength and it is easy to generate the diffraction phenomenon which can effectively overcome the problem of parasitic reflection due to foams, residues, deposits, etc. When a speaker emits sound waves with a uniform change from a frequency *f_L_* to a higher frequency *f_H_* toward the surface and a microphone receives the corresponding echoes, the generated standing wave signals extracted in the oscilloscope can be used to calculate the height of the liquid level. Kumperščak and Završnik [25,26] used this idea to measure liquid levels. However, they both directly used observations to calculate the liquid level. In practice, if the measurements obtained by using a speaker and a microphone are not precise enough and if the effect of environmental noise is inevitable, then the deviation of the final measurement results will be unacceptable, which is the most common shortcoming in the present level measurement methods.

In our earlier work [27], we have used the Evidential Reasoning(ER) rule to deal with liquid level estimation with bounded noises, but the ER-based method only provides an initial idea for state estimation under the framework of DS evidence theory and only gives precise estimated results when the level length is less than 1.6 m. In order to improve the evidence fusion-based state estimate method, this paper introduces a new information source, Dempster combination rule and evidence dependence conceptions. We construct the state equation and observation equation based on the principle of level measurement using acoustic standing waves, and then use the proposed algorithm to estimate the frequencies of the standing waves, which can be translated into the liquid level height (0 m–10 m). Compared with the direct measurement method and Nassreddine’s method, the estimation results verify that our algorithm has obvious advantages and improves the level estimation accuracy. 

### 4.1. Acoustic Standing Wave Level Gauge 

**Figure 5:**
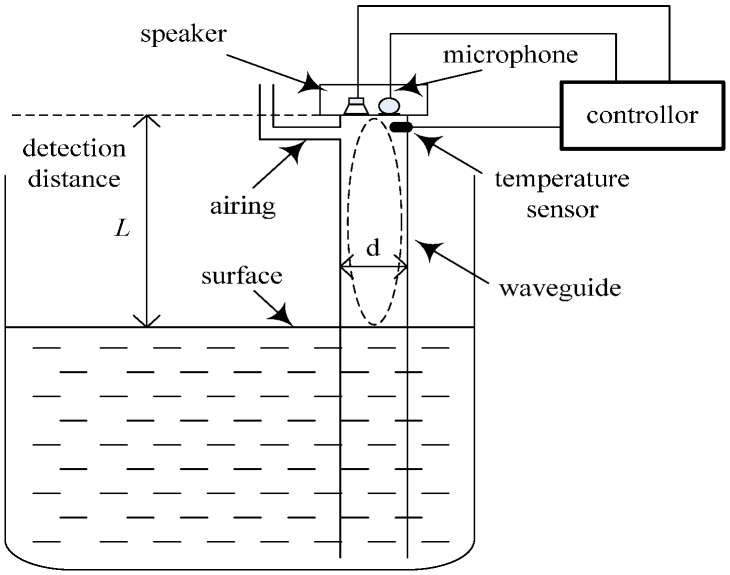
Structure of a level gauge.

The structure of an acoustic standing wave level gauge is shown in Figure 5, and mainly consists of a waveguide (a tube), a speaker, a microphone, a thermometer and a controller. When sound waves in the frequency range [*f_L_*,*f_H_*] generated by a signal generator (audio card and speaker) vertically propagate to a surface and echoes appear, superposition of both waves will generate standing waves. Here, *y*_1_ denotes the sound wave generated by speaker and *y*_2_ denotes the echo reflected by the surface:

(27) $$y_{1}=A\cos2\pi (Pt-\frac{L}{\lambda })$$

(28) $$y_{2}=A\cos2\pi (Pt+\frac{L}{\lambda })\;$$

The synthesis wave of *y*_1_ and *y*_2_ can be expressed as:

(29) $$y=2A\cos(\pi \frac{2L}{\lambda })\cos(2\pi Pt)$$

where *A* is the amplitude of sound wave, *P* is the frequency of sound wave and *L* is the distance from the top of the tube to the surface of liquid as shown in Figure 5. From Equation (29), we know that when *L* and *λ* have the following relation, the amplitude of synthesis wave reaches the maximum:

(30) $$L=n_{k}\frac{\lambda_{k}}{2}\;k=1,2,3,\cdots$$

In this case, this synthesis wave is defined as the standing wave and its wavelength is:

(31) $$\lambda_{k}=\frac{c}{f_{k}}=\frac{331.4+0.6T}{f_{k}}$$

where *λ_k_* is the wavelength of *k*th standing wave, *f_k_* is the frequency of *k*th standing wave (*k*th resonance frequency) in [*f_L_,f_H_*]. *c* is sound velocity, and *T* is temperature.

Substituting Equation (30) into Equation (31), we obtain:

(32) $$L=\frac{n_{k}(331.4+0.6T)}{2f_{k}}\;$$

where, *n_k_* is given as [28]:

(33) nk=fk(fk+1−fk)

and:

(34) $$n_{k+1}=n_{k}+1$$

Theoretically, in Equation (33), *f_k_*_+1_ − *f_k_* = *f_F_*, *f_F_* is the fundamental resonance frequency and *f_k_* = *n_k_ f_F_*, *n_k_* ∈ ℕ^+^ (the set of all positive integers) [26,28]. For example, if *L* = 9.6 m, *T* = 23.9 °C, and *n* = 1, then the fundamental resonance frequency can be calculated by (32):

(35) $$f_{F}=\frac{n(331.4+0.6T)}{2L}=18\mathrm{Hz}\;$$

If the frequency range [*f_L_,f_H_*] is [1000 Hz, 2500 Hz], then there are 82 resonance frequencies in this range, *k* = 1, 2,⋯, 82, and *n_k_* = 56,57,⋯,137. Consequentially, *f*_1_ = 56 × 18 Hz, *f*_2_ = 57 × 18 Hz,⋯, *f*_82_ = 137 × 18 Hz.

### 4.2. System Model

Firstly, we consider the resonance frequency as the estimated state and construct the corresponding state equation. If we can continuously collect the resonance frequency *f_k+_*_1_, then we have the following equations:

(36) $$L=\frac{n_{k+1}(331.4+0.6T)}{2f_{k+1}}\;$$

From Equations (32) and (36), obviously, we can get:

(37) $$\frac{n_{k+1}(331.4+0.6T)}{2f_{k+1}}=\frac{n_{k}(331.4+0.6T)}{2f_{k}}$$

(38) $$f_{k+1}=\;\frac{n_{k}+1}{n_{k}}f_{k}$$

Consequentially, we can establish the recursive linear state equation and observation equation, respectively:

(39) $$x_{k+1}=\frac{n_{k}+1}{n_{k}}x_{k}+v_{k}$$

(40) $$z_{k+1}=x_{k+1}+w_{k+1}$$

where *x_k_* = *f_k_*, *z_k_* is the observation of *f_k_*, *w_k_* and *v_k_* are independent noise sequences coming from speaker and microphone, respectively, satisfying the conditions:

(41) $$v_{k}=[v_{a},v_{b}]$$

(42) $$w_{k}=[w_{a},w_{b}]$$

The intervals [*v_a_,v_b_*] and [*w_a_,w_b_*] denote the boundaries of the state noise and observation noise, respectively. The state noise *v_k_* and observation noise *w_k_* can be expressed by possibility distributions *π_v_* and *π_w_* with the support intervals [*v_a_,v_b_*] and [*w_a_,w_b_*], respectively.

It should be noted that, in theory, *n_k_* in (39) should be taken as a positive integer. However, in practice, it can be only calculated by observations *z_k_* and *z_k_*_+1_ according to Equation (33). Because of observation imprecision, the calculated *n_k_* is commonly not a positive integer, so it should be approximated as:

(43) nk=‖zk(zk+1−zk)‖

where “‖•‖” denotes the operator that “round numbers to the nearest integer”.

### 4.3. Liquid Level Estimation Tests

**Figure 6:**
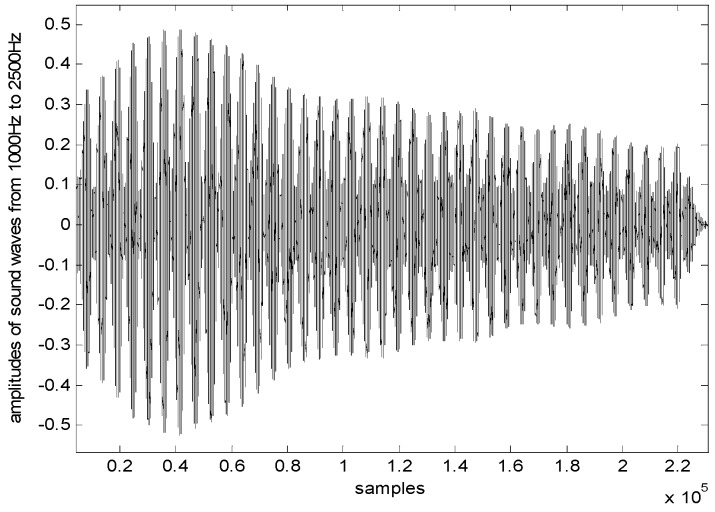
Waveform graph (*L* = 4.6 m).

**Figure 7:**
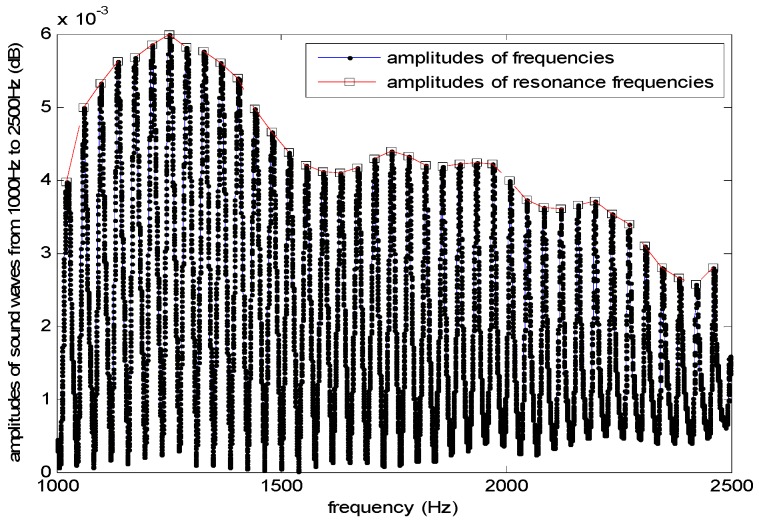
Resonance frequencies and amplitudes (*L* = 4.6 m).

In order to construct the level gauge in Figure 5, we use a low-precision microphone and speaker to emit and receive cosine sound waves, respectively, an electronic thermometer to collect temperature and a PVC tube with a diameter of *d* = 75 mm to transmit sounds. The estimated level *L* is the distance from the surface to the speaker platform. The controller transmits sine or cosine waves to drive the speaker to emit the signals vertically to the liquid surface. We use the software AUDIOSCSI (Brothers Studio, Shenzhen, China) which is based on an audio controller (82801HBM-ICH8M with sampling rate 44,100 Hz, Intel Corporation, Santa Clara, CA, USA) to generate sound waves. The frequencies of wave change with uniform speed from *f_L_* = 1000 Hz to *f_H_* = 2400 Hz in 5 s. Thus, the microphone receives the synthesis waves and sends them to the controller as shown in Figure 6 (*L* = 4.6 m). It can be seen that there are the frequencies of 39 adjacent standing waves collected by microphone in [1000 Hz, 2500 Hz]. Figure 7 shows the resonance frequency *f_k_* (*k* = 1,2,⋯, 39) extracted from the spectrum of the synthesis waves by the fast detecting algorithm in [28]. In this experiment, set liquid level distance *L* = 4.6 m, the ambient temperature is 26.5 °C and sound velocity is 347.3 m/s. The state equation and observation equation of resonance frequency are shown in Equations (39) and (40). 

**Figure 8:**
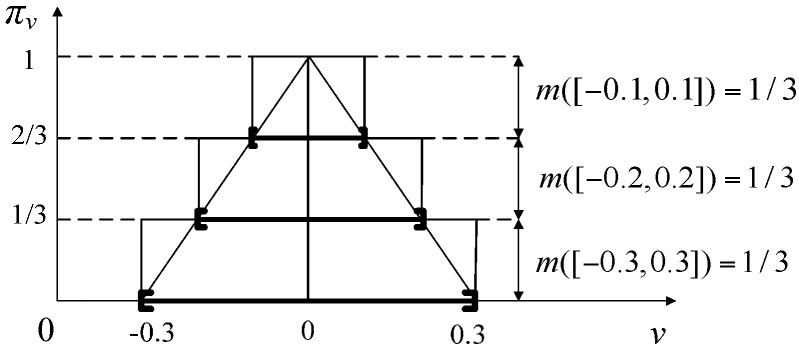
Probability distribution *π_v_* of state noise *v*.

For the state noise *v_k_*, we use a high-precision oscillograph (TPS2024, Tektronix, Shanghai, China) to receive the cosine sound waves emitted by the audio controller and speaker in range [1000 Hz, 2500 Hz] and calculate errors about 100 frequency points uniformly selected from 1000 Hz to 2500 Hz. As in [7], the bounds of *v_k_*, are taken to be plus or minus three times the standard deviation of errors. So the possibility distributions *π_v_* of *v_k_* can be constructed as in Figure 8. Where the expectation of *π_v_* is 0, standard deviation *σ_v_* is 0.1, then the support intervals [*v_a_,v_b_*] = [−0.3, 0.3], mode *v_c_*= 0. Set α_0_ = 0, α_1_ = 1/3, α_2_ = 2/3, we can get three nested closed intervals and their BBAs to approximate *π_v_* as in Figure 8. 

Furthermore, in Step 1 of the proposed algorithm in Section 3.2, discounting *m* at rate *ε_v_* = 0.05 and approximating as the closed interval [*v_c_ −* 100*σ_v_, v_c_ +* 100*σ_v_*] = [−10, 10], we can construct the evidence $\left({ℱ}_{k}^{v},m_{k}^{v}\right)$ of *v_k_* according to Equation (26) as shown in Table 1.

**Figure 9:**
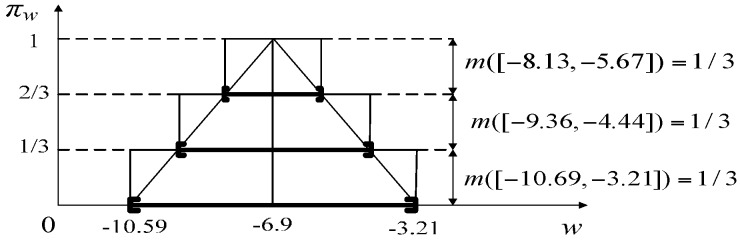
Probability distribution *π_w_* of observation noise *w*.

The observation noise *w_k_* is mainly related to the microphone and the fast detecting algorithm. Firstly, we extract observation values of resonance frequencies in range [1000 Hz, 2500 Hz] about 30 level values uniformly selected from *L* = 1.3 m to *L* = 10.6 m by the fast detecting algorithm. Secondly, we calculate the errors between the theoretically correct values (true values) and observation values. In the same way, the possibility distributions *π_w_* of *w_k_* and the corresponding closed intervals and their BBAs can be constructed as in Figure 9, where, *σ_w_* = 1.23, *w_c_* = −6.9, so [*w_a_,w_b_*] = [−10.59, −3.21]. Furthermore, discounting *m* at rate *ε_v_* = 0.05 and approximating as the closed interval [*w_c_* − 100*σ_w_,w_c_ +* 100*σ_w_*] = [−129.7, 115.7], we can construct the evidence $\left({ℱ}_{k}^{w},m_{k}^{w}\right)$ of *w_k_* shown in Table 2.

**Figure 10:**
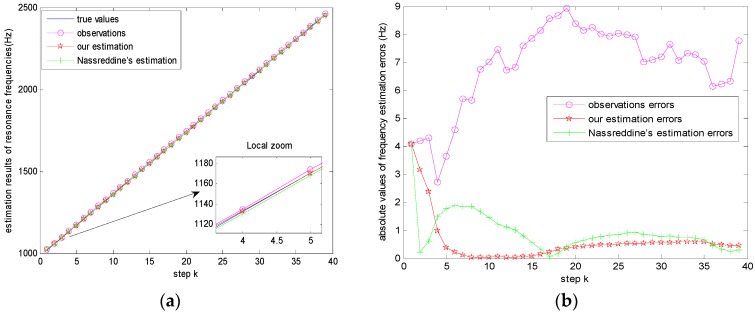
(**a**) Estimation results of resonance frequencies, (**b**) Absolute values of frequency estimation errors.

From Figure 7, it can be seen that the first observation value of resonance frequency *z*_1_ = 1023.3 Hz. According to Step 2) in Section 3.2, the first estimation result ${\hat{x}}_{1|1}$ is initialized as the real observation *z*_1_. After obtaining $\left({ℱ}_{1}^{v},m_{1}^{v}\right)$ and $\left({ℱ}_{1}^{w},m_{1}^{w}\right)$, our recursive algorithm presented in Section 3.2 can be used to estimate resonance frequencies at each step *k*. Figure 10a gives the estimation results of our method and Nassreddine’s method, together with the true values and observations (*z_k_*). Figure 10b gives the absolute errors between true values and the estimated values of our method, the estimated values of Nassreddine’s method, and *z_k_* respectively. It can be seen that the estimation accuracy and convergence of our method are better than those of Nassreddine’s method because of the focusing function of the proposed fusion procedure for dependent evidence.

**Figure 11:**
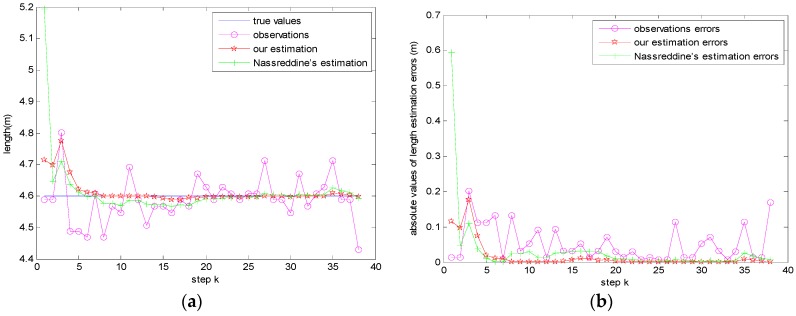
(**a**) Estimation results of level *L*, (**b**) Absolute values of length estimation errors.

Finally, we can calculate the estimate level *L* by Equation (32) according to the estimated resonance frequencies of our method, Nassreddine’s method, and *z_k_* respectively as shown in Figure 11a,b gives the corresponding absolute values of length estimation errors. Obviously, the more accurate the estimations of resonance frequencies are, the more accurate the estimations of level *L* are. As our method always provides more accurate estimations of resonance frequencies, it is therefore always superior.

More experiments are performed for different values of *L* to find the mean of absolute values of estimation errors and to show the efficacy of the proposed method as shown in Table 3. Here, for every values of *L*, from above to below, Table 3 gives in order the experiment results of our method, Nassreddine’s method, and direct measurement method (namely, substituting *z_k_* into (32)). 

It should be noted that the calculation complexity of our algorithm is relatively high, and meanwhile, with the increase of the measured length of level, the corresponding synthetic wave contains more and more resonance frequency points, so the CPU time will increase, so it needs a longer time (the hardware in this test: CPU E8400, CPU Clock Speed 3.00 GHz, RAM 2 GB). But to the situation that liquid level change relatively slowly, our method is applicable. Certainly, the rapid development of data processing capability of computer hardware will make the complexity less of an issue.

## 5. Conclusions

The subject of Nassreddine’s method is still interval analysis, it introduces evidence, namely belief-function to elaborate bounded noises. In detail, it gives the improved form of noise bounds (the triangular possibility distribution), here the error interval is extended to the evidence construction, namely many interval focal elements with the corresponding belief assignments. Obviously, the latter has more information than the former. Then, it still uses interval arithmetic and constraint-satisfaction techniques to propagate not only interval focal elements, but also its belief assignments, hence its performance is slightly better than that of pure interval propagation. However, the subject of our method is DS evidence theory and random set theory and introduces Dempster combination rule and evidence dependence conceptions. Although we still use Nassreddine’s evidence construction technique, the random set description of evidence and extension principle of random set are used to obtain state evidence and observation evidence from the defined information sources and to propagate them in the system equations. The main contribution is to realize the fusion of the propagated evidence, in which the degree of dependence and the combination of dependent evidence are further considered. 

As a whole, compared with Nassreddine’s method, our method increases the state estimation accuracy. The application in liquid level estimation using an industrial level apparatus shows the efficacy of the proposed method. Certainly, it is worth noting that, in a given application, there are some constraint conditions such as the continuous, monotonic and invertible properties of state and observation equations, and state observability. When they cannot be satisfied, the computational burden will inevitably be increased because of the use of additional complex interval operation algorithms or matrix operation algorithms (for multidimensional states) in [21,22]. Hence fast operation algorithms should be studied in the further. On the other hand, although the proposed procedure of evidence fusion can make the masses focus to those interval focal elements that contain the system state, so as to get the accurate estimation results, how to further evaluate the convergence of fusion using available theories is still a problem worthy studying which will promote the usage of evidence theory in state estimation. 

## Figures and Tables

**Table 1:** Evidence of state noise.

${ℱ}_{k}^{v}$	[−0.1, 0.1]	[−0.2, 0.2]	[−0.3, 0.3]	[−10, 10]
$m_{k}^{v}$	0.3167	0.3167	0.3167	0.05

**Table 2:** Evidence of observation noise.

${ℱ}_{k}^{w}$	[−8.13, −5.67]	[−9.36, −4.44]	[−10.59, −3.21]	[−129.7, 115.7]
$m_{k}^{w}$	0.3167	0.3167	0.3167	0.05

**Table 3:** Experimental results for different values of *L.*

No	True *L* (m)	*T* (°C)	Runtime (s)	*Mean Error* (m)	No	True *L* (m)	*T* (°C)	Runtime (s)	*Mean Error* (m)
1	1.3	27	1.81	0.0126	6	5.6	26.5	20.11	0.018
			0.88	0.016				4.81	0.0374
			-	0.238				-	0.0661
2	2.1	26.5	2.49	0.0254	7	6.6	26.5	23.57	0.0238
			1.33	0.0364				5.61	0.0436
			-	0.0441				-	0.088
3	2.6	26.5	7.81	0.0144	8	7.6	26.5	27.94	0.0299
			2.05	0.0297				6.77	0.0530
			-	0.0591				-	0.1060
4	3.6	26.5	9.62	0.0141	9	8.6	23.9	31.23	0.0216
			2.34	0.0312				7.41	0.0456
			-	0.0468				-	0.1295
5	4.6	26.5	16.07	0.0160	10	9.6	23.9	35.15	0.0435
			3.81	0.0337				8.36	0.0732
			-	0.0552				-	0.1624

## Appendix A

We first generalize the definition of evidence energy as shown in Proposition A1.

**Proposition** **A1.** *Suppose E = (ℱ_x_,m) is a body of evidence whose focal elements are the closed intervals. Energy of the evidence E can be defined by:*

(A1) $$\mathrm{En}\prime (E)=\sum_{\begin{array}{c} i=1\\ {}[x_{i}^{-},x_{i}^{+}]\neq \Theta \end{array}}^{n(E)}\frac{m([x_{i}^{-},x_{i}^{+}])}{d([x_{i}^{-},x_{i}^{+}])/\underset{i}{\min}(d([x_{i}^{-},x_{i}^{+}]))}$$

*where [x_i_^+^, x_i_^-^] denotes interval focal element, d([x_i_^-^, x_i_^+^]) means interval width and n(E) is the number of interval focal elements. For example, if E = (ℱ_x_,m), A*_1_ *= [−0.3, 2.6], m(A*_1_*) = 0.3; A*_2_ *=* [0.3,1.9]*, m(A*_2_*) = 0.7, mind([x_i_^-^, x_i_^+^]) = 1.6, then:*

(A2) $$\mathrm{En}\prime (E)=\frac{0.3}{2.9/1.6}+\frac{0.7}{1.6/1.6}=0.8655$$

*It can be proved that En′(E) meets the three conditions of the evidence energy: (1) if m(Θ) = 1, then En′(E) = 0 and E represents no useful information; (2) if* $d([x_{i}^{-},x_{i}^{+}])=\underset{i}{\min}(d([x_{i}^{-},x_{i}^{+}]))$ *and m(Θ) = 0, then En′(E) = 1 and E contains the maximum useful information; (3) En′(E) ∈ [0, 1].*

**Proof.** For mass function satisfying $\sum_{[x_{i}^{-},x_{i}^{+}]\subseteq \Theta }m([x_{i}^{-},x_{i}^{+}])=1$ and $d([x_{i}^{-},x_{i}^{+}])/\underset{i}{\min}(d([x_{i}^{-},x_{i}^{+}]))\geq 1$, there are four cases to discuss: Case 1: If *m*(Θ) = 1 and the mass of other focal elements is zero, then En′(*E*) = 0, namely, the evidence does not provide any information. So, the Condition (1) holds; Case 2: If *m*(Θ) = 0 and any interval focal element [*x_i_^+^,x_i_^-^*] meets that $d([x_{i}^{-},x_{i}^{+}])/\underset{i}{\min}(d([x_{i}^{-},x_{i}^{+}]))=1$, then En′(E) = 1, namely, the evidence provides maximum useful information. The Condition (2) holds; Case 3: If *m*(Θ) = 0 and not all of the interval focal elements [*x_i_^+^,x_i_^-^*] meet that $d([x_{i}^{-},x_{i}^{+}])/\underset{i}{\min}(d([x_{i}^{-},x_{i}^{+}]))=1$, then En′(*E*)∈(0,1); Case 4: If 0 < *m*(Θ) < 1, then En′(*E*)∈(0,1); From Case 1 to Case 4, the Conditions (3) can be proved. Let *E*_1_ = (ℱ*_x_*,*m_x_*) and *E*_2_ = (ℱ*_y_*,*m_y_*) be two pieces of evidence with closed interval focal elements. If some focal elements of *E*_1_ and *E*_2_ be induced by the same information sources, then the energy of the intersection of the two pieces of evidence can be described by:

(A3) $$\mathrm{En}\prime (E_{1},E_{2})=\sum_{\begin{array}{l} ij=1\\ D_{ij}\neq \emptyset \end{array}}^{\left|\{D_{ij}\}\right|}\frac{m(D_{ij})}{d(D_{ij})/\min\{\underset{x_{i}}{\min}\{d([x_{i}^{-},x_{i}^{+}]\},\underset{y_{j}}{\min}\{d([y_{j}^{-},y_{j}^{+}])\}\}}$$

where *D_ij_* is the focal elements induced by the same sources, {*D_ij_*} is the set of these focal element, |{*D_ij_*}| is the number of them. Consequentially, the degree of dependence between *E*_1_ and *E*_2_ can be obtained by Definition 5:

(A4) $$D\prime (E_{1},E_{2})=\frac{2\mathrm{En}\prime (E_{1},E_{2})}{\mathrm{En}\prime (E_{1})+\mathrm{En}\prime (E_{2})}$$

and the dependency coefficient between *E*_1_ and *E*_2_ can be redefined as:

(A5) $$R_{12}\prime =\frac{1}{2}D\prime (E_{1},E_{2})\frac{\mathrm{En}\prime (E_{2})}{\mathrm{En}\prime (E_{1})}$$

(A6) $$R_{21}\prime =\frac{1}{2}D\prime (E_{1},E_{2})\frac{\mathrm{En}\prime (E_{1})}{\mathrm{En}\prime (E_{2})}$$

*E*_1_ and *E*_2_ can be modified by $R_{12}\prime$ and $R_{21}\prime$ respectively to obtain the corresponding two pieces of independent evidence *E*_1_′ and *E*_2_′, their BBA functions are given by:

(A7) $$m_{1}\prime ([x_{i}^{-},x_{i}^{+}])=\left\{ \begin{array}{ll} m_{1}([x_{i}^{-},x_{i}^{+}])(1-R_{12}\prime ), & [x_{i}^{-},x_{i}^{+}]\neq \Theta \\ 1-\sum_{[x_{i}^{-},x_{i}^{+}]\prime \subset \Theta }m_{1}\prime ([x_{i}^{-},x_{i}^{+}]) & [x_{i}^{-},x_{i}^{+}]=\Theta \end{array}\right.$$

(A8) $$m_{2}\prime ([y_{j}^{-},y_{j}^{+}])=\left\{ \begin{array}{ll} m_{2}([y_{j}^{-},y_{j}^{+}])(1-R_{21}\prime ), & [y_{j}^{-},y_{j}^{+}]\neq \Theta \\ 1-\sum_{[y_{j}^{-},y_{j}^{+}]\subset \Theta }m_{2}\prime ([y_{j}^{-},y_{j}^{+}]) & [y_{j}^{-},y_{j}^{+}]=\Theta \end{array}\right.$$

After obtaining BBAs of *E*_1_′ and *E*_2_′, we can use Dempster combination rule to fuse *E*_1_′and *E*_2_′, namely, to fuse the dependent evidence *E*_1_ and *E*_2_ indirectly. ☐

Although Wu, Yang and Liu [17] gave the definition of the energy of the intersection as in Equation (3), this conception of intersection is obscure, namely, *D_ij_* and *m*(*D_ij_*) are rarely clearly defined. Nevertheless, in our method, the two pieces of dependent evidence are $\left({\hat{ℛ}}_{k+1}^{x},{\hat{\rho }}_{k+1}^{x}\right)$ and $\left({ℛ}_{k+1|k}^{x},\rho_{k+1|k}^{x}\right)$, according to the interval operations (extension principles and combination rule) used to generate them, $\left\{D_{ij}\right\}={\hat{ℛ}}_{k+1}^{x}\cap {ℛ}_{k+1|k}^{x}$ and $m(D_{ij})={\hat{\rho }}_{k+1}^{x}(D_{ij})=\rho_{k+1|k}^{x}(D_{ij})$.

For example, if the focal elements of the new fusion evidence $\left({\hat{ℛ}}_{k+1}^{x},{\hat{\rho }}_{k+1}^{x}\right)$ can be given as: *A*_1_ = [−0.30, 2.60], *m*(*A*_1_) *=* 0.3; *A*_2_ = [0.30, 1.90], *m*(*A*_2_) *=* 0.6, *A*_3_ = [0.32, 1.93], *m*(*A*_3_) *=* 0.1; the focal elements of the state estimation evidence $\left({ℛ}_{k+1|k}^{x},\rho_{k+1|k}^{x}\right)$ can be given as: *A*_1_ = [0.21, 3.50], *m*(*A*_1_) *=* 0.1, *A*_2_ = [0.41, 1.61], *m*(*A*_2_) *=* 0.3, *A*_3_ = [0.30, 1.90], *m*(*A*_3_) *=* 0.6, then Dij=[0.30,1.90], $m(D_{ij})=0.6$. From Equation (A1), obviously, we can get $\mathrm{En}\prime ({\hat{ℛ}}_{k+1}^{x},{\hat{\rho }}_{k+1}^{x})$ = 0.8655, $\mathrm{En}\prime ({ℛ}_{k+1|k}^{x},\rho_{k+1|k}^{x})$ = 0.7865, then the energy of the intersection of the two pieces of evidence can be calculated by Equation (A3):

(A9) $$\mathrm{En}\prime ({\hat{ℛ}}_{k+1}^{x},{\hat{\rho }}_{k+1}^{x}),({ℛ}_{k+1|k}^{x},\rho_{k+1|k}^{x})=\frac{m([0.3,1.9])}{d([0.3,1.9]/\min\{1.6,1.2\}}=\frac{0.6}{1.6/1.2}=0.45$$

We can calculate the degree of dependence between the new fusion evidence and the state estimation evidence by Definition 5 and from Equation (A4), we can get:

(A10) $$D\prime (({\hat{ℛ}}_{k+1}^{x},{\hat{\rho }}_{k+1}^{x}),({ℛ}_{k+1|k}^{x},\rho_{k+1|k}^{x}))=\frac{2\times \mathrm{En}\prime (({\hat{ℛ}}_{k+1}^{x},{\hat{\rho }}_{k+1}^{x}),({ℛ}_{k+1|k}^{x},\rho_{k+1|k}^{x}))}{\mathrm{En}\prime ({\hat{ℛ}}_{k+1}^{x},{\hat{\rho }}_{k+1}^{x})+\mathrm{En}\prime ({ℛ}_{k+1|k}^{x},\rho_{k+1|k}^{x})}=\frac{2\times 0.45}{0.8655+0.7865}=0.5448$$

The new fusion evidence is denoted as *E*_1_, and the state estimation evidence is denoted as *E*_2_. From Equations (A5) and (A6), we can get the dependency coefficient between *E*_1_ and *E*_2_:

(A11) $$R_{12}\prime =\frac{1}{2}\times 0.5448\times \frac{0.7865}{0.8655}=0.2475,\;R_{21}\prime =\frac{1}{2}\times 0.5448\times \frac{0.8655}{0.7865}=0.2997$$

From Equations (A7) and (A8), we can calculate the BBAs of the corresponding *E*_1_′ and *E*_2_′ as $m_{1}\prime (A1)=0.4733$, $m_{1}\prime (A2)=0.4515$, $m_{1}\prime (A3)=0.0752$ and $m_{2}\prime (A1)=0.3697$, $m_{2}\prime (A2)=0.2101$, $m_{2}\prime (A3)=0.4202$*.* Finally, using the Dempster combination rule to fuse *E*_1_′ and *E*_2_′, we obtain state estimation evidence $\left({\hat{ℱ}}_{k+1|k+1}^{x},{\hat{m}}_{k+1|k+1}^{x}\right)$ as: *A*_1_ = [0.21, 2.60], *m*(*A*_1_) *=* 0.1749, *A*_2_ = [0.41,1.61], *m*(*A*_2_) *=* 0.0994, *A*_3_ = [0.30, 1.90], *m*(*A*_3_) *=* 0.1988, *A*_4_ = [0.30, 1.90], *m*(*A*_4_) *=* 0.1669, *A*_5_ = [0.41, 1.61], *m*(*A*_5_) *=* 0.0948, *A*_6_ = [0.30, 1.90], *m*(*A*_6_) *=*0.1890, *A*_7_ = [0.32, 1.93], *m*(*A*_7_) *=* 0.0278, *A*_8_ = [0.41, 1.61], *m*(*A*_8_) *=* 0.0157, *A*_9_ = [0.32, 1.90], *m*(*A*_9_) *=* 0.0315.
